# Basophils activated via TLR signaling may contribute to pathophysiology of type 1 autoimmune pancreatitis

**DOI:** 10.1007/s00535-017-1390-6

**Published:** 2017-09-18

**Authors:** Masato Yanagawa, Kazushige Uchida, Yugo Ando, Takashi Tomiyama, Takashi Yamaguchi, Tsukasa Ikeura, Toshiro Fukui, Akiyoshi Nishio, Yoshiko Uemura, Takayuki Miyara, Hiroyuki Okamoto, Souhei Satoi, Kazuichi Okazaki

**Affiliations:** 1 The Third Department of Internal Medicine, Division of Gastroenterology and Hepatology, Kansai Medical University, 2-5-1 Shinmachi, Hirakata, Osaka 573-1010 Japan; 2 Department of Pathology, Kansai Medical University, Hirakata, Japan; 3 The First Department of Internal Medicine, Division of Hematology, Respiratory Medicine and Rheumatology, Kansai Medical University, Hirakata, Japan; 4 Department of Dermatology, Kansai Medical University, Hirakata, Japan; 5 Department of Surgery, Kansai Medical University, Hirakata, Japan

**Keywords:** Autoimmune pancreatitis, Basophil, TLR, M2 macrophage

## Abstract

**Background:**

Pathophysiology of type 1 autoimmune pancreatitis (AIP) is still unclear. We previously reported that M2 macrophages might play an important role in type 1 AIP. Recently, it has been reported that basophils regulate differentiation to M2 macrophages. In this study, we investigated basophils from the pancreatic tissue and peripheral blood of individuals with type 1 AIP.

**Methods:**

By using immunohistochemistry, we investigated basophils in pancreatic tissue from 13 patients with type 1 AIP and examined expression of toll-like receptors (TLRs) by these cells. Additionally, we obtained peripheral blood samples from 27 healthy subjects, 40 patients with type 1 AIP, 8 patients with alcoholic chronic pancreatitis, 10 patients with bronchial asthma, and 10 patients with atopic dermatitis, and analyzed activation of basophils by stimulating them with ligands of TLR1–9. We also compared TLR expression in basophils from the tissue and blood samples.

**Results:**

Basophils were detected in pancreatic tissues from 10 of 13 patients with type 1 AIP. Flow cytometric analysis revealed that the ratios of basophils activated by TLR4 stimulation in type 1 AIP (9.875 ± 1.148%) and atopic dermatitis (11.768 ± 1.899%) were significantly higher than those in healthy subjects (5.051 ± 0.730%; *P* < 0.05). Levels of basophils activated by TLR2 stimulation were higher in seven type 1 AIP cases. Furthermore, stimulation of TLR2 and/or TLR4, which were expressed by basophils in pancreas, activated basophils in peripheral blood.

**Conclusions:**

Basophils activated via TLR signaling may play an important role in the pathophysiology of type 1 AIP.

## Introduction

Autoimmune pancreatitis (AIP) is characterized by chronic pancreatic inflammation caused by immune mechanisms [1, 2]. Steroid treatment is remarkably effective for AIP, which may be sometimes difficult to be differentiated from pancreatic carcinoma [3]. The first report of AIP was a case of chronic pancreatitis with hypergammaglobulinemia that was published in 1961 [4]. Thirty years later, this pathological syndrome was defined as lymphoplasmacytic sclerosing pancreatitis (LPSP) [5], and proposed to be referred to as “autoimmune pancreatitis” by Yoshida et al. in 1995 [6]. Hamano et al. revealed that serum IgG4 levels were elevated in patients with AIP [7], and this condition is currently classified as type 1 AIP. On the other hand, Notohara et al. reported a different pathological condition, which was defined as idiopathic duct-centric pancreatitis (IDCP), which is classified as type 2 AIP [8]. In 2011, the international consensus diagnostic criteria (ICDC) for AIP classified two subtypes of AIP, type 1 and type 2 [9]. Type 1 AIP is recognized as the pancreatic manifestation of IgG4-related disease (IgG4-RD), which involves various organs, including the pancreas, salivary glands, thyroid glands, lungs, bile ducts, kidneys, retroperitoneum, lymph nodes, and others [10–13]. In serology, most patients with type 1 AIP exhibit elevated serum IgG4 levels [7], and some of them show elevated serum IgE [14, 15]. Histological features of type 1 AIP include diffuse lymphoplasmacytic and eosinophilic infiltrates, storiform fibrosis, obliterative phlebitis, and the infiltration of a large number of IgG4-positive plasma cells [9]. Regulatory T cells and T helper 2 (Th2) cytokines, such as IL-4, IL-5, and IL-13, are considered to contribute to pathophysiology of type 1 AIP [16, 17]. We have previously reported that the number of regulatory T cells is increased in peripheral blood and infiltrated target organs of type 1 AIP [18]. This particularly concerns inducible T cell co-stimulator (ICOS)-positive regulatory T cells, which promote IgG4 production via IL-10, and ICOS-negative regulatory T cells, which induce fibrosis via transforming growth factor (TGF)-β [18]. Furthermore, the pathophysiology of type 1 AIP is characterized by Th2-dominant reaction, but its mechanism has not yet been elucidated [19]. Our latest report suggested that M2 macrophages play an important role in the development of type 1 AIP [20]. IL-33 produced by M2 macrophages also promotes Th2 cytokine production conferring Th2-dominant pathophysiology in IgG4-RD [21]. Additionally, numerous macrophages were identified in AIP-affected tissue [22]. On the other hand, basophils were found to be required for the transformation of inflammatory monocytes into M2 macrophages in allergic disease [23]. However, the role of basophils in type 1 AIP is not clear.

In this study, to clarify the involvement of basophils activated via the toll-like receptor (TLR) signaling pathway in type 1 AIP, we investigated infiltration of TLR-expressing basophils in pancreatic tissue and their reactivity upon TLR stimulation in patients with type 1 AIP.

## Methods

In our experiments, we initially sought to confirm the presence of basophils in pancreatic tissue and then investigated whether they expressed TLRs. Furthermore, we examined the responses of basophils to TLR stimulation using peripheral blood mononuclear cells from patients with type 1 AIP.

### Subjects

For immunohistochemical analysis, we examined pancreatic tissue samples from 13 patients with type 1 AIP (6 men and 7 women; mean age 66 years; range 56–76 years).

We also conducted immunofluorescence analysis in 10 of 13 cases. All cases were surgically treated at the Kansai Medical University Hospital between 2001 and 2013, and in all cases, pancreatic ductal adenocarcinoma was suspected before the operation. The patients were histopathologically diagnosed with type 1 AIP. For comparison, we also examined pancreatic tissue samples from 10 patients with alcoholic chronic pancreatitis (ACP). All the 10 ACP patients underwent surgical operation.

For flow cytometry analysis, we recruited 27 healthy subjects (17 men and 10 women; mean age 66 years; range 42–83 years), 40 patients with type 1 AIP, who had not received corticosteroid treatment (29 men and 11 women; mean age 65 years; range 44–83 years), 8 patients with ACP (6 men and 2 women; mean age 57 years; range 39–69 years), 10 patients with bronchial asthma, who did not receive oral medication (6 men and 4 women; mean age 52 years; range 39–74 years), and 10 patients with atopic dermatitis, who did not receive oral medication (8 men and 2 women; mean age 49 years; range 27–77 years). Patient profiles are listed in Table 1. There was no difference in gender distribution between type 1 AIP and healthy subjects. The mean ages of bronchial asthma patients and atopic dermatitis patients were lower than those of patients with type 1 AIP and healthy subjects (*P* < 0.05). However, ages of patients with type 1 AIP, ACP, and healthy subjects were not significantly different. The mean values of serum IgG4 levels in type 1 AIP patients were significantly higher than in other groups (*P* < 0.05; Table 1). The numbers of basophils in the peripheral blood were not different in patients with type 1 AIP, ACP, and healthy subjects. All patients with type 1 AIP were diagnosed with AIP by the ICDC [9].

**Table 1:** Demographics and baseline characteristics of the patients in the present study

Patients characteristic	Healthy subject (*n* = 27)	Type 1 AIP (*n* = 40)	Alcoholic chronic pancreatitis (*n* = 8)	Bronchial asthma (*n* = 10)	Atopic dermatitis (*n* = 10)
Gender (male/female)	16/11	29/11	6/2	6/4	8/2
Age (years, mean ± SE)	65.63 ± 10.43	65.03 ± 10.70	57.00 ± 3.82	51.70 ± 13.38*	49.00 ± 17.31*
Serum IgG4 levels (mg/dL, mean ± SE)	32.90 ± 4.15 (*n* = 13)	465.85 ± 67.08 (*n* = 40)*	48.30 ± 12.36 (*n* = 8)	37.43 ± 6.75 (*n* = 8)	124.00 ± 33.03 (*n* = 8)

There was no difference in gender distribution between type 1 AIP and healthy subjects. The mean ages of bronchial asthma patients and atopic dermatitis patients were lower than those of patients with type 1 AIP and healthy subjects (*P* < 0.05). However, ages of patients with type 1 AIP, ACP, and healthy subjects were not significantly different. The mean values of serum IgG4 levels in type 1 AIP patients were significantly higher than in other groups (*P* < 0.05)

*NS* no significant differences compared with healthy or other disease controls

* *P* < 0.05 significant differences compared with healthy controls

This study was approved by the ethics committee of the Kansai Medical University.

### Immunohistochemistry and antibodies

We used mouse antibodies specific to human basophils (2D7; Abcam, Cambridge, UK) in order to identify basophils by immunohistochemical staining. The fields of view with the highest density of basophils were evaluated, and the numbers of basophils in three different fields of 1 mm^2^ each were summed. Next, we examined the expression of TLRs on infiltrated basophils using double-immunofluorescence staining of pancreatic tissue from patients with type 1 AIP with mouse antibodies specific to human basophils and rabbit antibodies specific to human TLR1–9.

For immunohistochemical staining, 4-μm-thick formalin-fixed and paraffin-embedded sections were prepared, deparaffinized, and rehydrated using xylene and solutions with gradually decreasing concentrations of alcohol. Endogenous peroxidase activity was blocked for immunohistochemical sections using a treatment with 3% H_2_O_2_ in methanol for 10 min at room temperature. After washing in distilled water, the slides were treated with 0.05% trypsin (Abcam) in PBS (pH 7.3) for 1 h at room temperature to enhance antigenicity. All slides were incubated for 10 min in Protein Block Serum-Free (Dako, Kyoto, Japan). After protein blocking, the slides were incubated overnight at 4 °C with mouse primary antibodies specific to human basophils (2D7). The primary antibodies were dissolved in Signal Enhancer HIKARI (Nacalai Tesque, Kyoto, Japan). The slides were then incubated with secondary antibodies using a Chem Envision kit/HRP (Dako) following the manufacturer’s instructions. Finally, antibody binding was detected using 3,3′-diaminobenzidine (Dojindo, Kumamoto, Japan). Sections were counterstained with hematoxylin. Negative controls were evaluated by replacing the primary antibodies with similarly diluted non-immunized serum. Images were obtained with a light microscope (DP73; Olympus, Tokyo, Japan).

In the double-immunofluorescence method, the slides were incubated with mouse antibodies specific to human basophils (2D7) and rabbit antibodies specific to human TLR1–9 (TLR1: AbFrontier, Seoul, Korea; TLR2: Novus, Littleton, CO, USA; TLR3: Novus; TLR4: StressMarq Biosciences, Victoria, Canada; TLR5: AbFrontier; TLR6: AbFrontier; TLR7: AbFrontier; TLR8: AbFrontier; TLR9: GeneTex, Irvine, CA, USA) as the primary antibodies overnight at 4 °C. The slides were then incubated with Alexa Fluor 546 anti-mouse immunoglobulins and Alexa Fluor 488 anti-rabbit immunoglobulins (Molecular Probes, Eugene, OR, USA) as secondary antibodies for 1 h at room temperature, protected from light. After incubation with the primary and secondary antibodies, slides were mounted with VECTASHIELD Mounting Medium and 4′,6-diamidino-2-phenylindole (DAPI; Vector Laboratories, Burlingame, CA, USA) to counterstain nuclei and preserve fluorescence. Negative controls were evaluated by replacing the primary antibodies with similarly diluted non-immunized serum. Images were obtained with an immunofluorescence microscope (DP73; Olympus). Additionally, we were able to obtain both the pancreatic tissues and blood from 5 out of the 10 patients, whose samples were used for immunofluorescence analysis, so we examined the relationship of each TLR.

### Flow cytometry and antibodies

We analyzed the activation of basophils using Allergenicity Kits (Beckman Coulter Company, Brea, CA, USA). The analysis of whole-blood specimens collected with EDTA as an anticoagulant was performed according to the manufacturer’s instructions. Whole-blood specimens were stimulated with TLR1–9 ligands Pam_3_CSK4 (400 ng/mL, InvivoGen, San Diego, CA, USA), HKLM (10^8^ cells/mL, InvivoGen), poly:IC (50 μg/mL, Sigma, St. Louis, MO, USA), LPS (1 μg/mL, Sigma), FLA-ST (10 μg/mL, InvivoGen), FSL-1 (5 μg/mL, InvivoGen), Imiquimod (5 μg/mL, InvivoGen), ssRNA40/LyoVec (5 μg/mL, InvivoGen) or ODN2006 (10 μg/mL, AdipoGen, San Diego, CA, USA), respectively. First, we mixed 100 μL of whole-blood specimens, activation solution, and three color reagents (CRTH2-FITC: BM16, CD203c-PE: 97A6, and CD3-PC7: UCHT1) with 10 μL of a TLR ligand or PBS in each tube. Activation solution is an optimized calcium-enriched buffer to activate basophils in vitro. Then, we incubated each tube for 15 min at 37 °C in the atmosphere of 95% air/5% carbon dioxide, protected from light. After staining, we added 2 mL of Fix-and-Lyse solution, which included the Allergenicity Kit, to remove erythrocytes interference. We incubated the tubes for another 10 min at room temperature, protected from light. We then centrifuged the tubes for 5 min at 200 g and aspirated the supernatant twice. Finally, we re-suspended the cells in 0.3 mL of PBS with 0.1% formaldehyde and analyzed them using flow cytometry. Flow cytometric analysis was performed by FACS Calibur II (BD Biosciences, Franklin Lakes, NJ, USA).

**Fig. 1:**
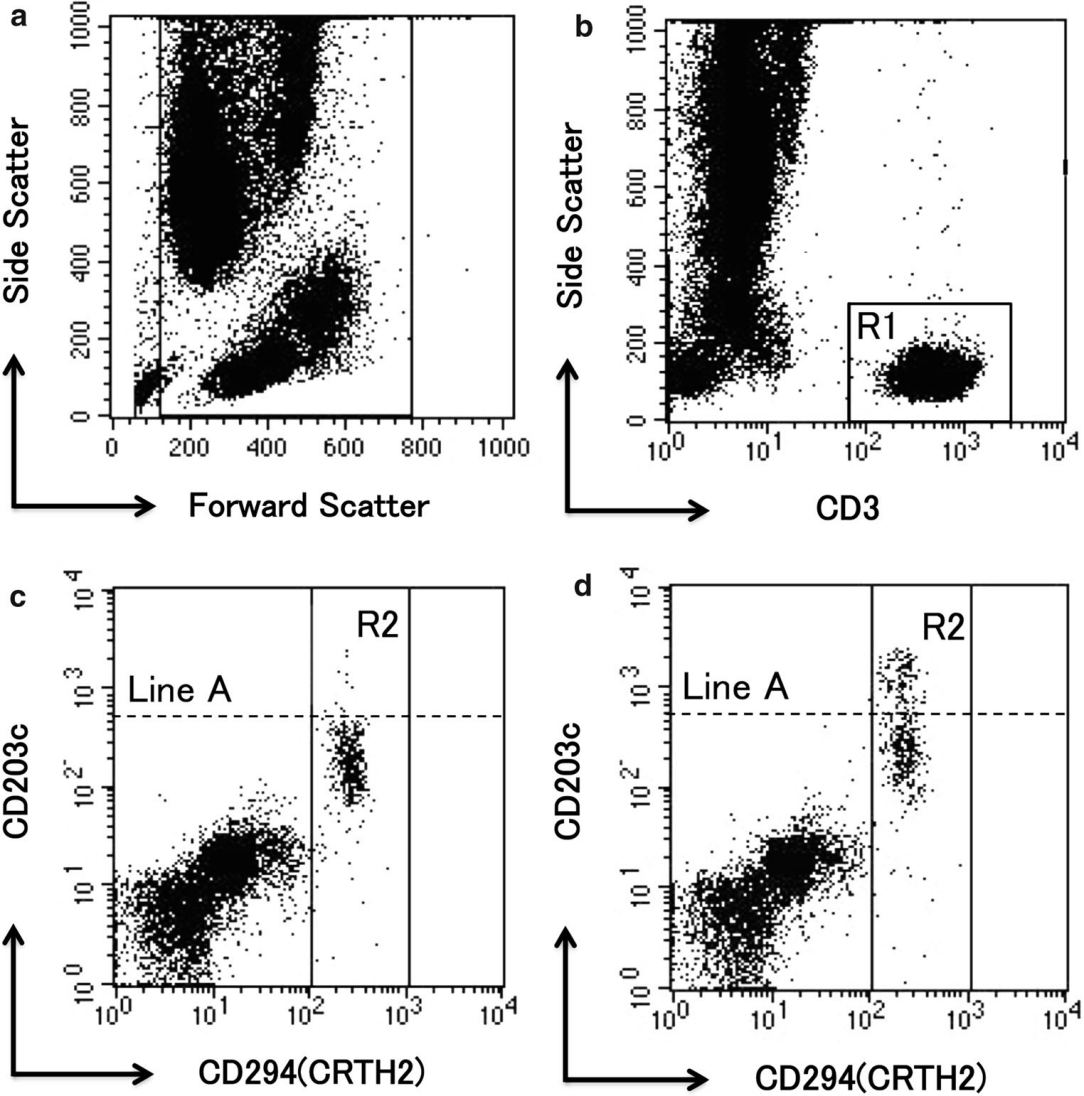
Identification of resting and activated basophils from peripheral blood by flow cytometry. All leukocytes were stained with CD3, CD294, and CD203c. All leukocytes formed discrete FSC/SSC populations after fixation and lysis (**a**). CD3-positive events (R1) were removed due to the isolation of lymphocytes and monocytes (**b**). Basophils were defined as CD294 (CRTH2)-positive events (R2) (**c**). CD203c is a specific marker of activated basophils. Line A was drawn so that 2–5% of basophils were included above it, and we defined them as activated basophils. We compared the percentage of basophils above line A before and after the stimulation (**d**). These experiments were performed using the Allergenicity Kit (Beckman Coulter Company, Brea, CA, USA)

We counted the ratio of activated basophils in accordance with the manufacturer’s instructions for the Allergenicity Kit (Fig. 1).

First, lymphocytes and monocytes were isolated, whereas CD3-positive events were excluded. Next, basophils were isolated as CD294 (CRTH2)-positive events from lymphocytes and monocytes. Finally, we drew line A so that 2–5% of basophils were included above it, and defined them as CD203c-positive basophils, because 2–5% of basophils are activated basophils in normal conditions. CD203c has been demonstrated to be a specific activation marker of basophils. Additionally, we compared the ratio of CD203c-positive basophils before and after TLR stimulation in each group.

### Statistical analysis

In immunohistochemistry and flow cytometry experiments, quantitative data are presented as the mean ± standard error of the mean (SE). The Tukey–Kramer test was used to compare quantitative values in flow cytometry experiments. Differences were considered to be statistically significant when the value of *P* was less than 0.05 (* *P* < 0.05).

## Results

### Identification of basophils in pancreatic tissue from patients with type 1 AIP

**Fig. 2:**
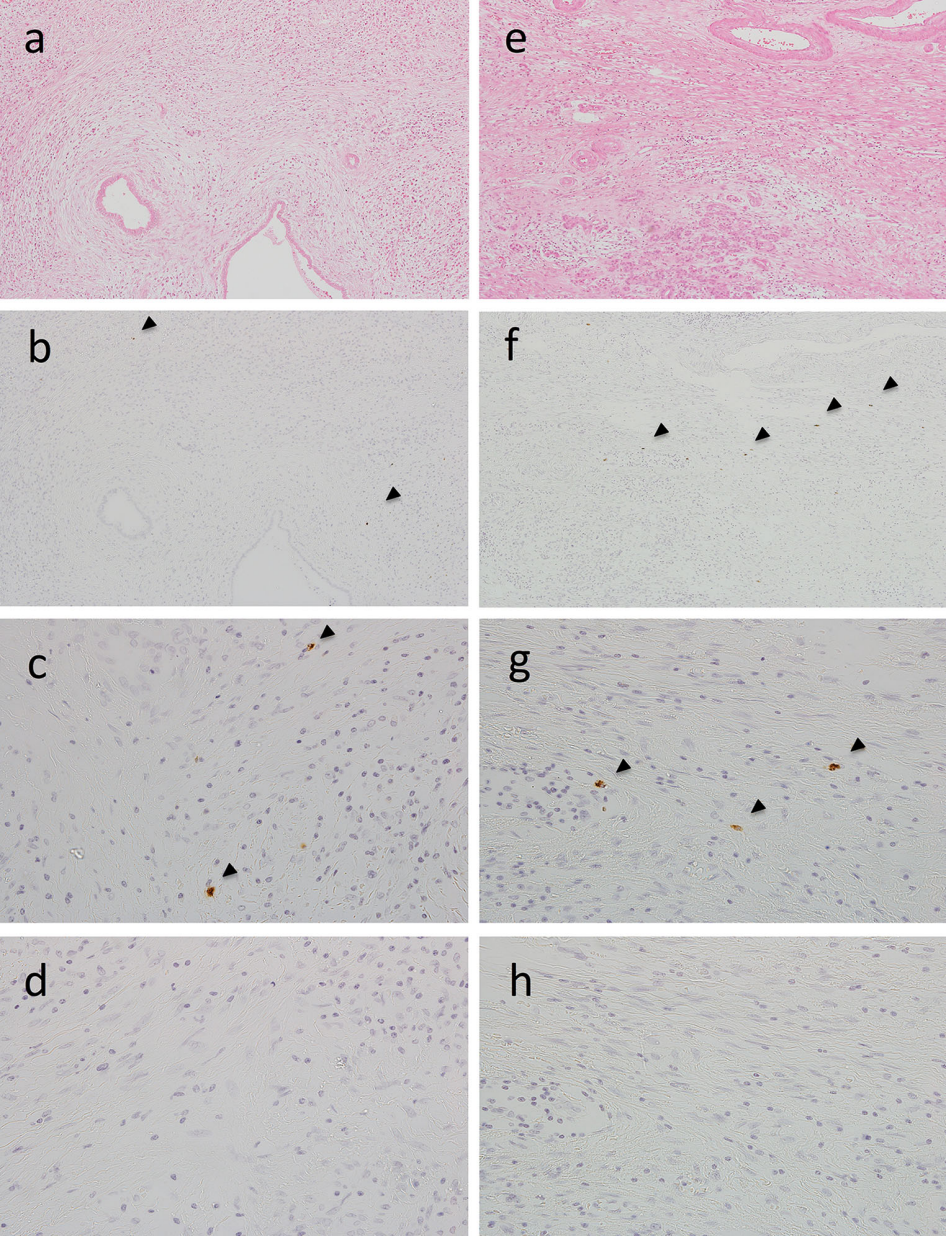
H&E staining (**a**, **e**), immunohistochemical findings for basophils [low magnification view, ×100] (**b**, **f**), [high magnification view, ×400] (**c**, **g**), and negative controls [high magnification view, ×400] (**d**, **h**) in pancreatic tissue samples of two cases with type 1 autoimmune pancreatitis (AIP). Infiltration of basophils was confirmed by using mouse anti-basophil antibodies (2D7)

**Fig. 3:**
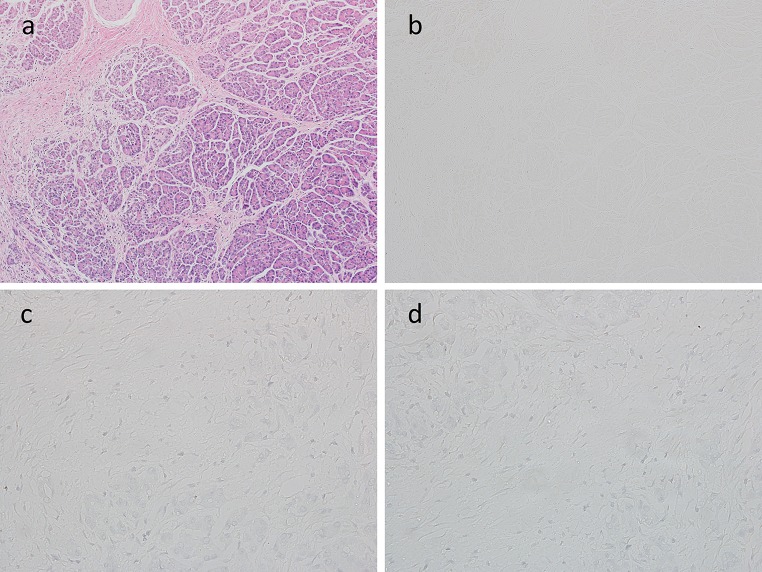
H&E staining (**a**), immunohistochemical findings for basophils [low magnification view, ×100] (**b**), [high magnification view, ×400] (**c**), and negative controls [high magnification view, ×400] (**d**) in a pancreatic tissue sample with alcoholic chronic pancreatitis (ACP)

**Fig. 4:**
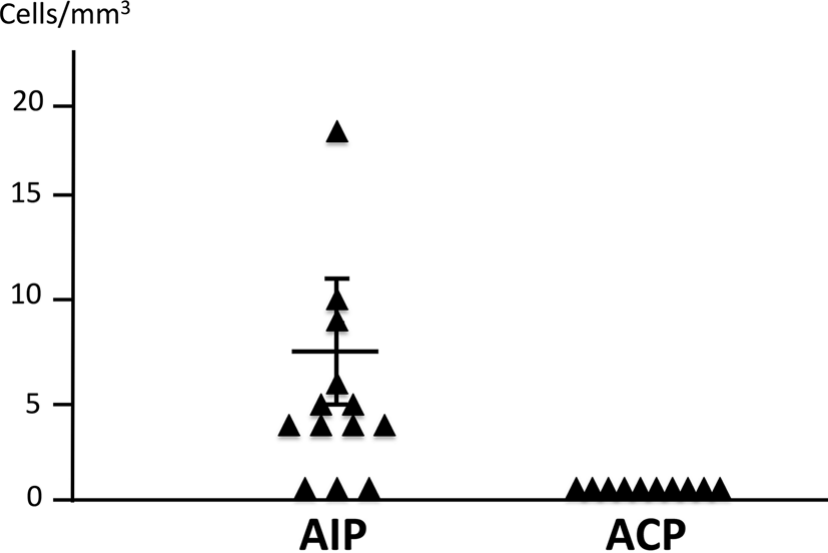
Comparison of the total number of basophils in three different 1-mm^2^ fields of view in pancreatic tissue samples from patients with type 1 AIP or alcoholic chronic pancreatitis (ACP). The average number of basophils in samples from patients with type 1 AIP was 8.615 ± 2.528 (*n* = 13). However, basophils were not detected in the pancreatic tissue of patients with ACP (*n* = 10)

Basophils (2D7-positive cells) were detected in the pancreatic tissue samples from 10 of 13 patients with type 1 AIP cases by immunohistochemistry (Fig. 2). In contrast, 2D7-positive cells were not detected in the pancreatic tissue from patients with ACP cases (Fig. 3). The average number of 2D7-positive cells was 8.615 ± 2.528, measured in three different 1-mm^2^ fields of view in samples from each of the 13 cases (Fig. 4).

### TLR expression by basophils from pancreatic tissue of patients with type 1 AIP

**Fig. 5:**
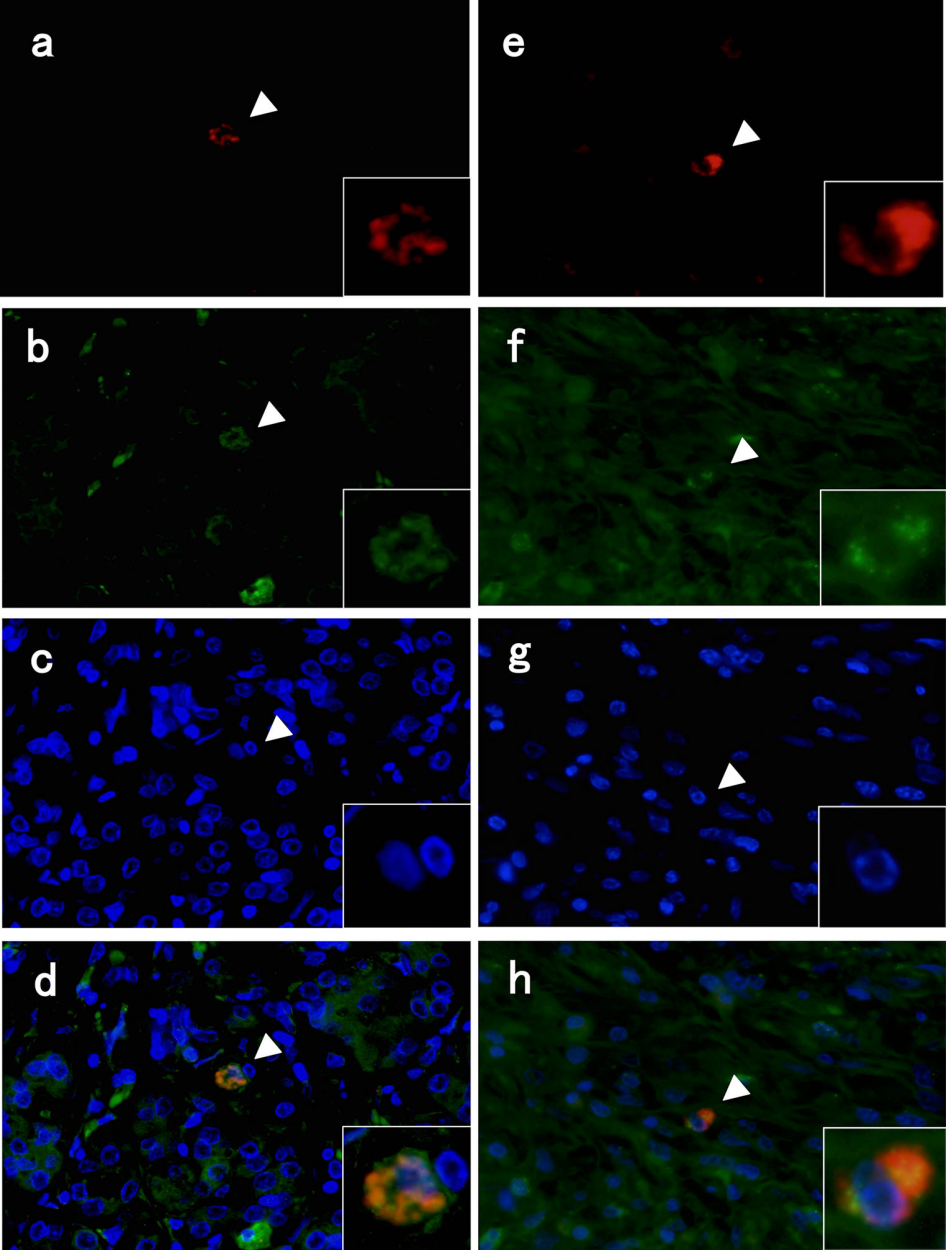
Double-immunofluorescence staining for basophils, TLR2, and TLR4 in pancreatic tissue of patients with type 1 autoimmune pancreatitis. Images show staining for basophils (2D7; red, **a**, **e**), TLR2 (green, **b**), TLR4 (green, **f**), and 4′,6-diamidino-2-phenylindole (DAPI, blue, **c**, **g**). Merged image of basophils and TLR2 (**d**)/TLR4 (**h**)

We identified 2D7-positive cells (red; Fig. 5a, e), TLR2-positive cells (green; Fig. 5b), and TLR4-positive cells (green; Fig. 5f), and examined them in the merged view (Fig. 5d, h), overlaid with DAPI nuclear staining (Fig. 5c, g). 2D7-positive cells expressed TLR2 in 2 of 10 cases, TLR4 in 6 of 10 cases, and both TLR2 and TLR4 in 2 of 10 cases. 2D7-positive cells were not detected among other TLR-positive cells (Table 2).

**Table 2:** TLR expression on basophils infiltrated into the pancreatic tissue with type 1 AIP

Case no.	Age	Gender	TLR1	TLR2	TLR3	TLR4	TLR5	TLR6	TLR7	TLR8	TLR9
1	63	M	−	+	−	−	−	−	−	−	−
2	71	F	−	+	−	−	−	−	−	−	−
3	78	F	−	+	−	+	−	−	−	−	−
4	67	M	−	+	−	+	−	−	−	−	−
5	67	M	−	−	−	+	−	−	−	−	−
6	56	M	−	−	−	+	−	−	−	−	−
7	57	F	−	−	−	+	−	−	−	−	−
8	60	F	−	−	−	+	−	−	−	−	−
9	60	F	−	−	−	+	−	−	−	−	−
10	71	M	−	−	−	−	−	−	−	−	−

### Relationship between TLR expression by basophils from pancreatic tissue and activation of peripheral blood basophils upon stimulation of TLR1–9

We examined the relationship between TLR expression by basophils from pancreatic tissue and activation of basophils in the peripheral blood by stimulating them with TLR1–9 ligands. We found that the levels of activated basophils in the peripheral blood were elevated by TLR2 stimulation in the two patients (case 1 and 2) that exhibited TLR2-positive basophils in the pancreatic tissue. In one case (case 3), which exhibited both TLR2- and TLR4-positive basophils in the pancreatic tissue, levels of activated basophils in the peripheral blood of this patient were elevated by only both TLR2 and TLR4 stimulation. In the two cases (case 5 and 6) that exhibited TLR4-positive basophils in the pancreatic tissue, levels of activated basophils in peripheral blood were elevated by TLR4 stimulation, but not other types of TLR (Table 3). Thus, in patients with type 1 AIP, stimulation of the TLR that is expressed on the basophils in the pancreatic tissue seems to activate circulating basophils.

**Table 3:** Relationship of the TLR expression on basophils in the peripheral blood and the pancreas from type 1 AIP

Case no.	Age	Gender	Serum IgG4 (mg/dL)	TLR expression on basophils in the pancreas	Activated ratio of basophils in the peripheral blood (%)	
					TLR2	TLR4
1	63	M	153	TLR2	60.1	3.9
2	71	F	119	TLR2	8.0	4.8
3	78	F	65.9	TLR2, 4	65.4	7.4
5	67	M	20	TLR4	5.0	9.6
6	56	M	1140	TLR4	3.0	10.2

Case no. is the same as those in Table 2. No. 4, 7–10 have not been tested by flow cytometry

In case 1 and case 2, which exhibited TLR2-positive basophils in the pancreatic tissue, levels of activated basophils in the peripheral blood of these patients were elevated by TLR2 stimulation. In case 3, which exhibited both TLR2- and TLR4-positive basophils in the pancreatic tissue, levels of activated basophils in peripheral blood were elevated by only both TLR2 and TLR4 stimulation. In case 5 and case 6, that exhibited TLR4-positive basophils in the pancreatic tissue, levels of activated basophils in peripheral blood were elevated by TLR4 stimulation, but not other types of TLR

### Ratios of basophils activated by TLR1–9 stimulation

**Fig. 6:**
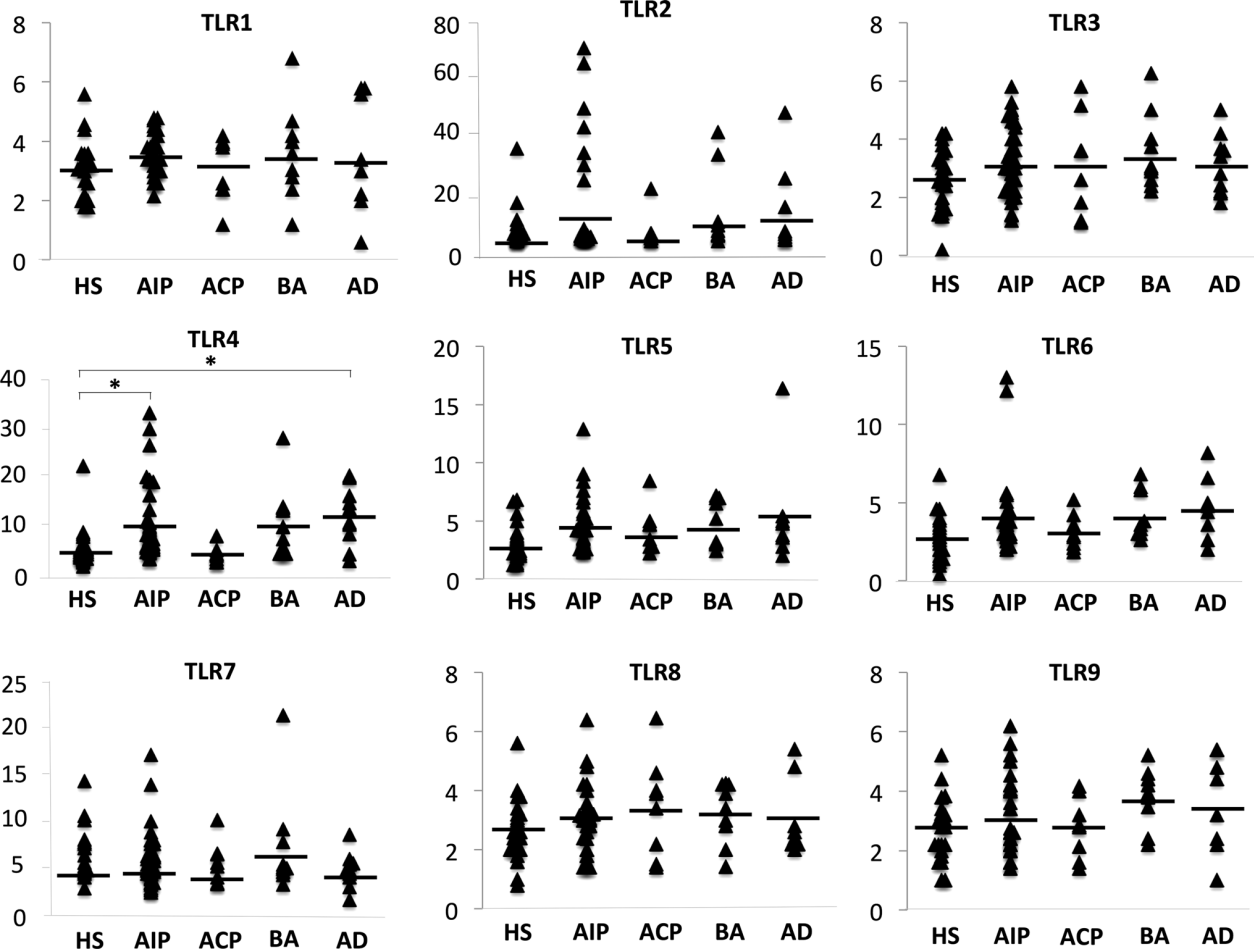
The ratio of basophils activated by the stimulation of TLR1–TLR9. We analyzed peripheral basophils in healthy subjects (*n* = 27), patients with type 1 autoimmune pancreatitis (AIP, *n* = 40), patients with alcoholic chronic pancreatitis (ACP, *n* = 8), patients with bronchial asthma (*n* = 10), and patients with atopic dermatitis (*n* = 10). Basophils were stimulated with the TLR1–9 ligands, Pam_3_CSK4 (400 ng/mL, InvivoGen, San Diego, CA, USA), HKLM (10^8^ cells/mL, InvivoGen), poly:IC (50 μg/mL, Sigma, St. Louis, MO, USA), LPS (1 μg/mL, Sigma), FLA-ST (10 μg/mL, InvivoGen), FSL-1 (5 μg/mL, InvivoGen), imiquimod (5 μg/mL, InvivoGen), ssRNA40/LyoVec (5 μg/mL, InvivoGen), or ODN2006 (10 μg/mL, AdipoGen, San Diego, CA, USA), respectively. Data are presented as the mean ± standard error of the mean. Statistical comparisons of quantitative data were carried out by the Tukey–Kramer test. Differences were considered to be significant when the value of *P* was less than 0.05 (*P* < 0.05)

In the absence of TLR stimulation, CD203c expression levels in healthy subjects (265.897 ± 34.449), patients with type 1 AIP (238.364 ± 17.130), ACP (235.84 ± 44.43), bronchial asthma (251.546 ± 50.610), and atopic dermatitis (194.674 ± 49.491) in mean fluorescence intensity (MFI) were not significantly different, and the ratio of activated basophils in healthy subjects (2.758 ± 0.144%), patients with type 1 AIP (3.167 ± 0.156%), patients with ACP (2.256 ± 0.133%), bronchial asthma (3.489 ± 0.282%), and atopic dermatitis (2.946 ± 0.118%) were also not significantly different (data not shown). However, the ratios of basophils activated by TLR4 stimulation in the blood of patients with type 1 AIP (9.875 ± 1.148%) and atopic dermatitis (11.768 ± 1.899%) were significantly higher than that in healthy subjects (5.051 ± 0.730%; *P* < 0.05). Furthermore, the ratio of TLR4-activated basophils in patients with type 1 AIP were not significantly elevated compared to ACP, but tended to be elevated (*P* = 0.08). In 7 of the 40 patients with type 1 AIP, CD203c expression levels were elevated by TLR2 stimulation, although the effect did not reach statistical significance. In addition, CD203c expression levels following stimulation of other TLRs were not significantly different between the groups (Fig. 6).

## Discussion

In this study, we identified the presence of basophils in pancreatic tissue of patients with type 1 AIP. We also found that the ratio of basophils activated by TLR4 signaling in the peripheral blood of patients with type 1 AIP was significantly higher than that in healthy subjects. In addition, the ratio of basophils activated by TLR2 signaling was elevated in 7 of 40 cases of type 1 AIP (Fig. 6). Remarkably, we revealed that stimulation of the type of TLR that was expressed on infiltrated basophils in type 1 AIP-affected pancreatic tissue also activated circulating basophils (Table 3). On the other hands, basophils were not detected in the pancreatic tissue with ACP, and the ratio of basophils activated by TLR signaling in the peripheral blood of patients with ACP were not significantly different as compared with healthy subjects. Furthermore, the ratio of TLR4-activated basophils in patients with type 1 AIP tended to be elevated compared to ACP. Therefore, we speculated that basophils activated by TLR signaling were important in the development of type 1 AIP.

Basophils comprise less than 1% of human peripheral blood leukocytes and can live only for a few days in the non-activated condition [24]. However, it has been shown that 10-times higher levels of Th2 cytokines such as IL4 and IL13 are immediately produced compared with those in lymphocytes, even when a small percentage of basophils are activated [25, 26]. They are generally not present in normal tissue and become recruited to affected tissue sites only under certain conditions, for example, during allergic reactions [27]. It has been shown that basophils act as initiators of inflammatory cell recruitment during the progression of IgE-mediated chronic allergic inflammation [28]. It has also been reported that basophils in skin lesions from atopic dermatitis were detected in approximately 60% of patients; however, the cell density was low as compared with patch-tested lesions [29]. Patients with type 1 AIP have frequent complications of allergic conditions, such as bronchial asthma or allergic dermatitis, characterized by elevated serum IgE and eosinophilia [21, 30]. In this study, we identified basophils in pancreatic tissue samples from 10 of 13 patients with type 1 AIP (Fig. 4), but the cell density was low. This finding indicates that pathophysiology of type 1 AIP may be similar to that of an allergic disease, such as atopic dermatitis. We, therefore, speculate that basophils infiltrating in the tissues might play an important role in the Th2-dominant condition of type 1 AIP.

Basophil-derived IL-4 is considered to be involved in the recruitment of monocytes and their differentiation to M2 macrophages in allergic skin [23]. Macrophages are composed of at least two distinct groups of M1 and M2 phenotypes. M1 macrophages elicit an inflammatory responses and play a central role in host defense against bacterial and viral infections, whereas M2 macrophages play roles in anti-inflammatory responses, reactions to parasitic infections, as well as in tissue repair and remodeling [31]. M2 macrophages induced by IL-4 are involved in fibrosis as they produce IL-10 and CCL18 [32]. Notohara et al. reported that CD163-positive spindle-shaped macrophages contribute to LPSP manifestations, such as storiform fibrosis in type 1 AIP [33]. We previously reported that macrophages (especially CD163-positive cells), stimulated via TLR signaling pathways, might play an important role in pancreatic tissue from patients with type 1 AIP [20]. Furthermore, the number of M2 macrophages in salivary glands of individuals with IgG4-RD was significantly higher than that in healthy subjects, and the distribution of IL-10 and CCL18 closely paralleled that of M2 macrophages [34]. These findings suggest that basophils induce M2 macrophages, which, in turn, may contribute to fibrosis in the pancreatic tissue affected by type 1 AIP.

It is known that Th2 cytokine production is elevated in pancreatic tissue of type 1 AIP patients, but the mechanism of this phenomenon has not yet been clarified [35]. It was recently demonstrated that endogenous IL-33 promoted Th2 cytokine production and induced allergic inflammation during allergic airway inflammation [36]. IL-33 produced by M2 macrophages promoted Th2 cytokine production via IL-33 receptor activation and contributed to Th2-dominant pathophysiology in IgG4-RD [37]. Another study revealed that basophils rapidly produced large amounts of IL-4 in response to various stimuli, including activation by ligands of TLRs, in innate-type allergy, and could induce differentiation of Th2 cells without cross-linkage of FcεRI [38]. On the basis of these reports and our present data, we speculate that basophils are important cells in Th2-dominant pathophysiology of type 1 AIP.

Additionally, we identified the presence of TLR2- and/or TLR4-positive basophils in pancreatic tissue from patients with type 1 AIP (Table 2). We could confirm that basophils express TLR2 or TLR4 in this study, but some researchers reported that antigen presenting cells such as dendritic cells, macrophages, B cells, and T cells express TLR2 or TLR4 [39–41]. The presence of basophils expressing different TLRs (TLR2 and/or TLR4) in the pancreatic tissue suggests that pathophysiology of type 1 AIP may be caused by a heterogeneous inflammatory condition.

There was no difference of the clinical data (blood test results, location of pancreatic swelling, or extent of involvement of other organs) between the TLR2-positive group and TLR4-positive group in AIP patients. There was also no correlation between the ratio of basophils activated by TLR signaling and serum IgG4 and IgE levels (data not shown). However, Watanabe et al. reported that basophils activated via TLR2 or TLR4 signaling enhanced the production of IgG4 through a BAFF-mediated signaling pathway in IgG4-RD [42]. On the other hand, there was no correlation between serum IgG4 levels and the number of IgG4-positive cells in AIP patients (data not shown). Paik et al. also reported that there was no correlation between serum and tissue IgG4 levels [43]. Basophils that infiltrated into pancreatic tissue of patients with type 1 AIP may also contribute to IgG4 production.

TLRs are pattern recognition receptors that recognize pathogen-associated molecular patterns (PAMPs) or damage-associated molecular patterns (DAMPs). PAMPs are associated with microbial pathogens, whereas DAMPs are linked to cell components, which are released during cell damage or death, to induce the innate immune response [43]. It is well known that PAMPs derived from abundant gut bacteria are transferred to the serum and bone marrow, where they promote systemic innate immunity [44]. It is also possible that DAMPs activate basophils in pancreatic tissue from patients with type 1 AIP. DAMPs of TLR2 and TLR4 include biglycan, high-mobility group box protein 1 (HMGB1), heat shock protein 70, fibronectin, and others [45]. They have been shown to play roles in the induction of experimental pancreatitis [46]. HMGB1, released by necrotic acinar cells, induces tissue injury and inflammation via TLR4 activation [47, 48]. As a result of acinar cell necrosis, HMGB1 may activate basophils via TLR4 in pancreatic tissue of type 1 AIP patients. HMGB1 and self-nucleic acid complexes are also involved in the development of chronic inflammatory disease [49, 50]. Thus, excessive immune reactions mediated by endogenous TLR ligands may be involved in the development of type 1 AIP. The difference in the function of basophils activated via TLR2 and TLR4 signaling is still unclear. Basophils activated via TLR2 and/or TLR4 signaling may participate in Th2 immune response and elimination of endogenous debris [51, 52, 53]. In the future, it will be necessary to clarify the differences in the properties of basophils activated via different TLRs in order to better understand pancreatitis pathophysiology.

In conclusion, our results demonstrate that together with M2 macrophages, basophils activated via TLR signaling may also play an important role in pathophysiology of type 1 AIP.

## Abbreviations

AIP: Autoimmune pancreatitis
LPSP: Lymphoplasmacytic sclerosing pancreatitis
IDCP: Idiopathic duct-centric pancreatitis
ICDC: International consensus diagnostic criteria
IgG4-RD: IgG4-related disease
ICOS: Inducible T cell co-stimulator
TGF-β: Transforming growth factor-β
TLR: Toll-like receptor
ACP: Alcoholic chronic pancreatitis
PAMPs: Pathogen-associated molecular patterns
DAMPs: Damage-associated molecular patterns
HMGB1: High-mobility group box protein 1
